# Do Neutrophil–Lymphocyte Ratio and Platelet–Lymphocyte Ratio Need to Be Stratified for Age and Comorbidities in COVID-19 Disease? A Subgroup Analysis of Two Distinct Cohorts over Disease Course

**DOI:** 10.3390/jcm13020605

**Published:** 2024-01-21

**Authors:** Nadya Kagansky, Yochai Levy, Anas Awar, Estela Derazne, Alexander Shilovsky, Dana Kagansky, Victor Chepelev, Evelina Mazurez, Ilia Stambler, Osnat Levtzion-Korach

**Affiliations:** 1 School of Medicine, Tel Aviv University, Tel Aviv 69978, Israel; 2 Shmuel Harofe Geriatric Medical Center, Be’er Ya’akov 70350, Israel; 3 Shamir Medical Center, Rishon Le-Zion 70300, Israel

**Keywords:** nursing homes, geriatric, frailty, old age, COVID-19, neutrophil-to-lymphocyte ratio, platelet-to-lymphocyte ratio

## Abstract

Several studies described neutrophil–lymphocyte ratio (NLR) and platelet–lymphocyte ratio (PLR) as markers of COVID-19 severity. In a recently published study, age and frailty affected NLR and PLR more than disease severity. The study compared two distinct cohorts. The first comprised older frailer patients positive for SARS-CoV-2, with mild or asymptomatic disease, admitted to designated COVID-19 departments in a large geriatric medical center (GMC). The second cohort comprised COVID-19 patients admitted to a large general hospital (GH) for symptomatic disease. This was a follow-up study comparing a subgroup of patients who had NLR and PLR values measured a week after admission. Only 100 of 177 patients in the original GMC cohort had a second NLR test compared to almost all (287 of 289) patients in the general hospital (GH) cohort. The subgroup baseline characteristics did not change significantly from that of the original cohort. Disease symptoms were more prevalent in the GH cohort. In the GMC group, the median second NLR rose from 3.9 to 4.6, while in the GH cohort, the NLR value dropped from 3.5 to 2.8, enhancing the NLR differences between the groups. Smaller changes were observed in the second PLR. These results strengthen the prior results that age and frailty seem to have a stronger impact on NLR and PLR than disease severity.

## 1. Introduction

The coronavirus disease 2019 (COVID-19) global pandemic crisis was responsible for the deaths of millions and still affects the lives of billions worldwide [1]. Countries are now adapting to live with COVID-19, while trying to improve clinical care for COVID-19 patients [2]. In Israel, during the pre-vaccination era of the COVID-19 pandemic, a strict policy was executed including screening of close contacts of COVID-19-positive patients, screening of residents in long-term care facilities where a COVID-19 carrier was found and, for periods of time, routine screening of residents for COVID-19. All subjects found positive for the virus needed to enter a week of isolation [3,4,5]. This policy led to the identification of many asymptomatic or pre-symptomatic COVID-19 patients both at home and in long-term care facilities. To enable isolation and as an attempt to prevent outbreaks in long-term care facilities, designated COVID-19 departments were opened, mainly in geriatric hospitals. These departments admitted mainly older adults with mild or asymptomatic disease, who were unable to complete the isolation period at home, or were in long-term care residents and in need of continuous care [4]. Designated COVID-19 departments in general hospitals were reserved for more symptomatic COVID-19 patients. In these settings and while the pandemic is still prevalent worldwide, identification of rapid and reliable clinical biomarkers that indicate disease severity may be crucial.

Neutrophiles and platelets obtained in a simple blood count test usually rise as a result of systemic inflammation, [6,7] while the lymphocytes count may decline [8]. Severe COVID-19 infection is described as a highly inflammatory disease. The proposed mechanism is a rapid and severe cytokine storm [6] resulting from an unregulated activation of the innate immune system and the release of damage-associated molecular patterns following tissue injury. These patterns include a heightened production of low-density neutrophils (LDNs) and the formation of neutrophil extracellular traps (NETs), resulting in elevated NLR and unfavorable outcomes in individuals diagnosed with severe COVID-19 [9]. For these reasons, in severe COVID-19 patients, the neutrophils count is expected to be high and the lymphocytes count low, making the neutrophils–lymphocytes ratio (NLR) and the platelets–lymphocytes ratio (PLR) attractive markers of disease severity.

NLR was described to be an easily obtained inflammatory indicator with a prognostic role in several medical conditions including infections [10,11,12], malignancies [13,14,15], cardiovascular diseases [16], influenza [17], and more. Several studies including meta-analyses have found elevated admission NLR in COVID-19 patients to be associated with poor outcomes. However, the definition of elevated NLR values in the studies was inconsistent, which made its clinical significance questionable. PLR was also described as an easily obtained inflammatory marker. Its role in COVID-19 patients is more ambiguous [18,19,20].

In a recently published study [21], we described admission NLR and PLR in two COVID-19 patient groups, one comprising frail older adults with mild and mostly asymptomatic disease admitted to designated COVID-19 departments of a geriatric medical center (GMC) and the other a group of patients with a disease considered moderate or severe admitted to a large hospital in Israel. Our results showed significantly higher NLR and PLR in the GMC group, indicating that age, frailty, and comorbidities play a more significant role in NLR and PLR values than COVID-19 severity.

Most patients in the geriatric medical center (GMC) were asymptomatic upon admission, raising the question whether the results were due to the disease stage and timing of the test (before the clinical presentation of the disease). In this study, we present a subgroup analysis of patients who had consecutive NLR and PLR blood tests during their admission in COVID-19 departments.

## 2. Materials and Methods

The design, eligibility, and baseline characteristics were previously published [14]. This was a follow-up retrospective, observational, two-cohort comparative study. The first cohort comprised patients positive for SARS-CoV-2, with mild or asymptomatic disease, admitted to designated COVID-19 departments in a large skilled GMC. The second group included patients admitted to an affiliated large hospital for symptomatic COVID-19. Inclusion criteria to this sub-study were second NLR and PLR tests about a week after the admissions test (4–10 days). High NLR values of over 6.5 were previously reported to corelate with severe COVID-19 disease [22]. The number of patients with NLR over 6.5 was low in our cohorts. Normal cut-off values for NLR and PLR are a matter of debate and, therefore, we chose a dichotomic cut-off value of 2.15 as relatively high for NLR and a value of 110 as relatively high for PLR. These values are slightly higher than the median values published in a large study by Wang et al. who suggested normal NLR values from 1.5 in young males to 1.76 in females over 80 years old and normal PLR values of up to 109 [23].

Data were retrieved from electronic medical records (EMRs) and included age, gender, demographic variables, comorbidities, chronic medication, symptoms, and laboratory tests on admission and one week after admission. We evaluated the clinical changes of the participants that occurred from admission to one week later, assuming that some in the GMC group were pre-symptomatic on admission. We aimed to examine the association of COVID-19 patients with NLR and PLR and their change over time as the disease progresses.

### Statistical Analysis

A high NLR value was predetermined at 2.15. Assuming proportions of low NLR (<2.15) of 10% and 25% in GMC and GH cohorts, respectively, in the second test and using an α = 0.05 test of proportion difference (2-sided using binomial enumeration), we achieved a power (1-β) = 0.871. We used an independent sample proportion test to compare between changes in GMC- and GH-group proportions from the first to the second NLR and PLR tests (from high to low or from low to high). Because the interval measurement variables (age, height, weight, Body Mass Index, number of children, number of total diseases, total number of medications, NLR, PLR, and selected laboratory data) were not normally distributed, we presented median and 25th–75th percentile values in tables and utilized the Mann–Whitney test to compare between GMC and GH groups. The presence of disease and symptoms at admission were compared between the 2 groups with Fisher’s exact test, and the McNemar test was used to evaluate changes that occurred from admission to one week later. We compared categorical demographic characteristics between the 2 groups with a Chi-square test or Fisher’s exact test in 2 × 2 tables. Spearman’s correlation was calculated between NLR and PLR differences (second–first test result) and selected laboratory changes. Statistical analysis was performed with IBM SPSS Statistics for Windows, version 29 Armonk, IBM Corp, New York, NY, USA. A two-sided *p*-value ≤ 0.05 was considered statistically significant.

## 3. Results

Between March and September 2020–2021, data were collected from 177 patients admitted to the COVID-19-designated department in the GMC and 289 COVID-19 patients admitted to GH care. Only 100 patients had a second NLR test in the GMC group compared to almost all (287 of 289) patients in the GH group. The subgroup baseline characteristics are described in Table 1 and did not change significantly from those of the original cohort.

In the GMC subgroup, patients were older (by over three decades); most of them were in need of continuous supervisions due to limitations in physical or mental status and had significantly more comorbidities and medications. Almost half of the patients in the GMC cohort were long-term care residents compared to only 7% in the GH cohort. On admission, all common disease symptoms were more prevalent in the GH group and only 2.1% of patients were asymptomatic compared to 62% in the GMC subgroup (Table 2).

Only 9 patients in the GMC group developed new symptoms at the time of the second blood test, while symptoms resolved for 11 patients. No changes in symptoms were statistically significant (Table 3). The GH group had significantly more asymptomatic patients on the second test, implying disease resolution in many cases. For example, out of 197 patients presenting with fever on admission in the GH group, only 6 patients still suffered from fever a week from admission. No patients in the GH group developed a new fever.

Except for monocytes%, results from the blood tests were significantly different between the GMC and GH cohorts, as shown in Table 4. Also, the changes from the first to the second test differ in NLR, hemoglobin, monocytes%, lymphocytes, platelets, creatinine, and CPR between the two cohorts.

**Figure 1:**
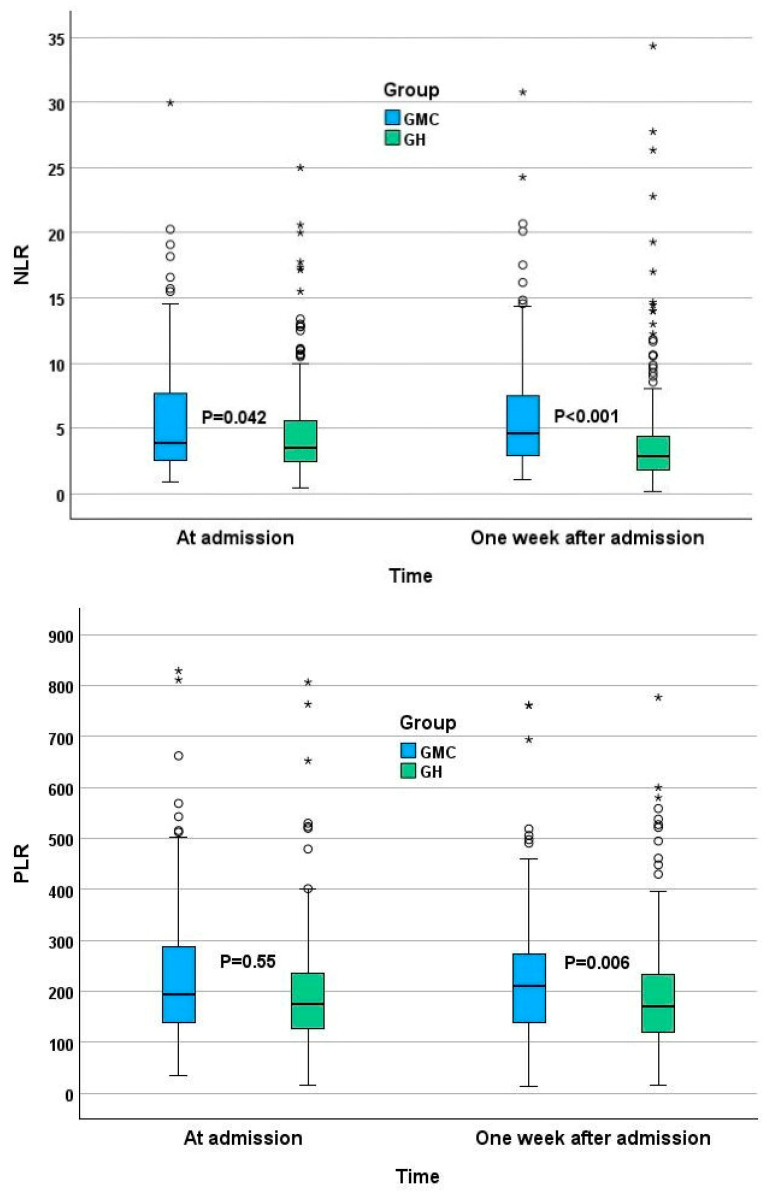
NLR and PLR at admission and a week after admission. * Horizontal bars represent median, boxes—lower and upper quartile, whiskers—minimal and maximal values. Circles represent outliners, stars represent extreme values.

NLR and PLR values are presented in Figure 1. In the GMC group, the median second NLR rose from 3.9 to 4.6, while the GH cohort showed an opposite change with the NLR value dropping from 3.5 to 2.8. While smaller changes were observed in the second PLR, the direction of the change was the same as in the NLR, making the second PLR significantly higher in the GMC group. The majority of patients in both groups had high NLR (higher than 2.15) and PLR (higher than 110) values in both tests (Table 5).

## 4. Discussion

In a prior publication [14], we described the NLR and PLR differences in two distinct patient groups admitted to COVID-19 departments, suggesting NLR and PLR are influenced by age, frailty, and comorbidities more than by disease severity. This deduction was based only on admission NLR and PLR, leaving the question as to whether these differences were the result of early versus later disease stage at the time of admission. In this follow up sub-study, we found larger differences in consecutive NLR and PLR tests a week after admission.

Although the length of hospital stay was longer in the GMC group, only 100 of 177 patients had second blood test results during their hospitalization, compared to the vast majority (287/289) in the GH group. This is another indication of the differences in disease severity between the groups. The NLR changes in the second test enhanced the differences between the groups. This is probably a result of different disease stages upon admission. Most patients admitted to the GMC had asymptomatic disease and were found to be COVID-19-positive on a screening test. The hospitalized patients in the GH were probably close to the peak of the disease, explaining the decline in the NLR and PLR values in the later test. This hypothesis is supported by the resolution of symptoms in many of the HG patients (Table 3) in the second test and also by the decline in the CRP values found in the GH group (Table 4). The majority of the patients in the GMC group remained asymptomatic and were discharged after finishing a mandatory isolation period. We believe the results strengthen our prior conclusion by emphasizing the differences between the groups. A fair assumption from our results is that even close-to-peak NLR values in the GH group were lower compared to those in the GMC group comprising older adults with multimorbidity but with a milder disease (Table 4). Though PLR changes were not as prominent as those in the NLR, they also demonstrated the same results of higher second PLR in the GMC group and a lower second PLR in the GH group, strengthening this conclusion.

In our study, the vast majority of patients in both cohorts had NLR values higher than 2.15 and significantly higher means of both NLR and PLR than suggested normal values. These results imply that NLR and PLR are probably elevated in COVID-19 patients. The differences between groups in NLR and PLR in both tests were higher and more prominent than the results described in different age groups of a healthy population [15]. This may be a result of differences in the immune system response at different ages’ health and functional status and the differences in comorbidities.

NLR and PLR values in this study were lower than described in most prior publications on COVID-19 patients [11] in concordance with the mild or asymptomatic disease in our cohorts. Prior studies have shown a relationship between NLR values and COVID-19 severity. In a recent meta-analysis, Parthasarathi et al. demonstrated that admission NLR predicts both severity and mortality in COVID-19 patients, and an NLR > 6.5 is associated with significantly greater odds of mortality [22]. The study stated that NLR values of patients were not influenced by age or major comorbidities; however, the results showed that severe COVID-19 patients were generally older and had a greater number of comorbidities. Furthermore, when examining the patients’ characteristics within the studies, they were dramatically different from those of our cohort. The vast majority of patients in the meta-analysis were of Chinese origin, frailty and performance status were usually not described, and the patients were significantly younger. For example, the largest trail in the analysis on 1859 of the 7332 patients assessed in the meta-analysis had a median age of less than 60 years [24]. In the present study, we demonstrated an unequivocal difference between the two cohorts whose main differences were age (median of 85Y compared to 52Y), functional status, and comorbidities. Age and frailty were both found to play a role in COVID-19 severity [25,26], and several studies have demonstrated elevated NLR in frail patients [27,28,29,30]. We found another study on COVID-19 patients that also demonstrated an independent association between NLR and frailty [31]. However, we found no large study assessing NLR and PLR prognostic value in COVID-19 patients stratified to age and frailty, leaving a possibility for causality (due to confounding variables).

This study has several limitations. Frailty was not assessed using a validated test; this was assessed in the introduction and in a prior study. Functional status, comorbidities, poly-pharmacy and older age all have a significant correlation to frailty [32,33]. We believe the described major differences between the cohorts make the assumption of a frailer cohort a fair one. Only 100 patients out of 177 in the GMC had a second blood test; this may cause a selection bias. It is plausible that a second test was taken in more severe cases; however, it is highly unlikely this would have a major impact on the results. Patients in the GMC still had mild disease and the majority were discharged back to their prior accommodation. Furthermore, most patients who developed severe disease were hospitalized and, therefore, it is unlikely that patients who remained in the GMC had a more severe disease than the GH cohort. We did not have access to new medications given throughout the hospitalization. Some medications, such as corticosteroids, may influence NLR values. Corticosteroids were part of the treatment protocol in severe COVID-19 patients during most of the study period. However, due to the low number of severe COVID-19 cases, this should not have a significant effect on median values. Due to the mild disease course of most patients, the prognostic NLR and PLR values in severe disease are beyond the scope of this study. It is possible that severe COVID-19 patients have significantly elevated NLR and PLR; however, it is highly questionable whether the differences in NLR and PLR values, which grew more significant in a consecutive test, would fade in severe disease. These differences should be addressed in future studies.

## 5. Conclusions

A comparison of second NLR and PLR tests in the described distinct cohorts demonstrated greater differences than the already significant differences of prior tests. These results suggest that age, comorbidities, and frailty have a stronger impact on NLR and PLR than disease severity. Although NLR and PLR appear to be elevated in COVID-19 patients, without adjusting for age and frailty, their use as a marker for disease severity may be limited. Larger-scale studies are needed to evaluate adjusted NLR and PLR and their changes in different chronic and acute diseases. This study does not exclude the possibility that extremely high NLR and PLR values may still play a role in acute settings.

## Figures and Tables

**Table 1:** Baseline characteristics of the study cohorts.

	GMC		GH		
	N	Median (25th–75th)	N	Median (25th–75th)	*p*
Age	100	84.5 (78.0–91.0)	287	52.0 (42.0–58.0)	<0.001
BMI *	37	24.1 (21.7–26.6)	271	27.8 (24.7–31.6)	<0.001
	**N**	**%**	**N**	**%**	*p*
**Sex**					0.002
Male	36	36.0	156	54.4	
Female	64	64.0	131	45.6	
**Residence**					<0.001
Home	51	51.5	267	93.0	
Nursing Home	48	48.5	20	7.0	
**Total number of diseases**					<0.001
0	0	0.0	98	34.1	
1	1	1.0	49	17.1	
2	2	2.0	58	20.2	
3	8	8.0	33	11.5	
4	9	9.0	25	8.7	
≥5	80	80.0	24	8.4	
Heart failure	25	25.0	10	3.5	<0.001
Chronic kidney disease	23	23.0	11	3.8	<0.001
Dementia	30	30.0	19	6.6	<0.001
Depression	17	17.0	9	3.1	<0.001
Asthma–COPD	9	9.0	24	8.4	0.837
CVA	21	21.0	2	0.7	<0.001
Diabetes mellitus	44	44.0	69	24.0	<0.001
Anemia	29	29.0	10	3.5	<0.001
Hypertension	82	82.0	76	26.5	<0.001
Pressure Ulcers	11	11.0	2	0.7	<0.001
Coronary Disease	28	28.0	23	8.0	<0.001
PVD	5	5.0	1	0.3	0.005
Hyperlipidemia	47	47.0	68	23.7	<0.001
Chronic Liver Disease	6	6.0	7	2.4	0.107
Hypothyroidism	22	22.0	14	4.9	<0.001
**Total number of medications**					<0.001
0	0	0.0	130	45.3	
1–3	5	5.0	73	25.4	
4–6	28	28.0	49	17.1	
6–9	67	67.0	35	12.2	
Ace-Arb-inh	42	42.0	53	18.5	<0.001
B-blockers	52	52.0	39	13.6	<0.001
Insulin	18	18.0	27	9.4	0.029
Ca-blockers	36	36.0	27	9.4	<0.001
Vitamin D	33	33.0	11	3.8	<0.001
Antiplatelets	31	31.0	42	14.6	0.001
Levothyroxine	19	19.0	12	4.2	<0.001
Anticoagulants	43	43.0	15	5.2	<0.001
Antipsychotics	35	35.0	21	7.3	<0.001
Antidepressants	31	31.0	33	11.5	<0.001
Corticosteroids	8	8.0	11	3.8	0.109

* Abbreviation: BMI, body mass index; COPD, chronic obstructive pulmonary disease; CVA, cerebral vascular accident; PVD, peripheral vascular disease.

**Table 2:** Symptoms on admission in the two cohorts.

	GMC		GH		
	N	%	N	%	*p*
Anosmia	0	0.0	13	4.5	0.025
Diarrhea	1	1.0	31	10.8	0.001
Fatigue	4	4.0	153	53.3	<0.001
Headache	0	0.0	45	15.7	<0.001
Fever	16	16.0	197	68.6	<0.001
Cough	11	11.0	146	50.9	<0.001
Anxiety	1	1.0	4	1.4	1.000
Delirium	2	2.0	1	0.3	0.165
Dyspnea	26	26.0	132	46.0	0.001
Abdominal pain	2	2.0	32	11.1	0.004
Change of appetite	2	2.0	48	16.7	<0.001
No symptoms	62	62.0	6	2.1	<0.001

**Table 3:** Patients’ distribution of symptoms on admission and a week later.

GMC Symptoms *	+/+	+/−	−/+	−/−	McNemar P
Anosmia	0	0	0	100	NA
Diarrhea	0	1	2	97	1
Fatigue	1	3	1	95	0.625
Headache	0	0	0	100	NA
Fever	4	12	4	80	0.077
Cough	7	4	4	85	1
Anxiety	1	0	0	99	1
Delirium	1	1	0	98	1
Dyspnea	17	9	6	68	0.607
Abdominal pain	1	1	2	96	1
Change of appetite	2	0	0	98	1
Any symptoms	27	11	9	53	0.824
GH Symptoms *	**+/+**	**+/−**	**−/+**	**−/−**	**McNemar P**
Anosmia	0	13	1	273	0.002
Diarrhea	1	30	2	254	<0.001
Fatigue	12	141	7	127	<0.001
Headache	1	44	3	239	<0.001
Fever	6	191	0	90	<0.001
Cough	24	122	1	140	<0.001
Anxiety	0	4	1	282	0.375
Delirium	0	1	0	286	NA
Dyspnea	15	117	6	149	<0.001
Abdominal pain	1	31	1	254	<0.001
Change of appetite	1	47	2	237	<0.001
Any symptoms	89	192	1	5	<0.001

* +/+ positive on admission/positive at 1 week; +/− positive on admission/negative at 1 week; −/+ negative on admission/positive at 1 week; −/− negative on admission/negative at 1 week.

**Table 4:** Changes between the first and second blood tests in the study cohorts.

		First Test					Changes from First to Second Test		
		GMC		GH		M−W P	GMC	GH	M−W P
		N	Median (25th−75th)	N	Median (25th−75th)		Median (25th−75th)	Median (25th−75th)	
NLR		100	3.86 (2.52–7.67)	286	3.52 (2.44–5.56)	0.042	0.20 (−1.70 to 2.09)	−0.08 (−1.85 to 0.02)	0.006
PLR		95	195 (139–302)	285	175 (128–237)	0.039	−3.29 (−61.77 to 52.61)	0 (−41.36 to 31.43)	0.736
Hemoglobin	g/dL	100	11.5 (10.3–13.4)	286	13.4 (12.5–14.5)	<0.001	−0.5 (−1.5 to 0.2)	0 (−0.8 to 0.2)	0.006
WBC	10^3^/µL	100	7.8(5.9–10.6)	286	5.7 (4.20–7.40)	<0.001	0.8 (−2.1 to 2.5)	0 (−0.6 to 1.4)	0.730
Monocytes%	%	100	8.2 (5.9–10.1)	284	8.2 (6.2–10.5)	0.281	−0.4 (−2.2 to 1.3)	0 (−1.0 to 2.1)	0.006
Neutrophil TN	10^3^/µL	100	5.7 (4.0–8.5)	286	3.9 (2.7–5.3)	<0.001	0.5 (−1.4 to 2.4)	0 (−0.8 to 0.7)	0.147
lymphocyte TN	10^3^/µL	100	1.3 (0.9–1.7)	287	1.0 (0.8–1.5)	0.001	0.0 (−0.4 to 0.4)	0.1 (0.0 to 0.6)	<0.001
Platelets	10^3^/µL	95	255 (187–334)	285	184 (143–237)	<0.001	6 (−47 to 65)	16 (0 to 120)	0.001
RDW	%	100	14.5 (13.6–16.0)	285	13.7 (13.1–14.5)	<0.001	0.2 (−0.3 to 0.7)	0 (−0.1 to 0.2)	0.070
Creatinine	mg/dL	99	1.0 (0.7–1.3)	281	0.8 (0.6–09)	<0.001	0 (−0.15 to 0.14)	−0.05 (−0.1 to 0)	0.003
CRP	mg/L	72	32 (17− 56)	275	53 (21–112)	0.005	0 (−16 to 35)	−3.62 (−61 to 0)	<0.001

Abbreviation: M–W, Mann–Whitney; NLR, neutrophil–lymphocyte ratio; PLR, platelet–lymphocyte ratio; WBC, white blood cells; TN, total number; CRP, c-reactive protein.

**Table 5:** High NLR (≥2.15) and PLR (≥110) in the study cohorts.

	GMC		GH		
	N	%	N	%	*p*
NLR ≥ 2.15	83	83.0	229	79.8	0.558
PLR ≥ 110	85	85.0	240	83.6	0.874

## Data Availability

The datasets presented in this article are not readily due to privacy and ethical restrictions.
